# Disabling chronic conditions in childhood and socioeconomic disadvantage: a systematic review and meta-analyses of observational studies

**DOI:** 10.1136/bmjopen-2014-007062

**Published:** 2015-09-03

**Authors:** Nicholas J Spencer, Clare M Blackburn, Janet M Read

**Affiliations:** Warwick Medical School, University of Warwick, Coventry, UK

**Keywords:** SOCIAL MEDICINE

## Abstract

**Objective:**

To determine the association of socioeconomic disadvantage with the prevalence of childhood disabling chronic conditions in high-income countries.

**Study design:**

Systematic review and meta-analyses.

**Data sources:**

6 electronic databases, relevant websites, reference lists and experts in the field.

**Study selection:**

160 observational studies conducted in high-income countries with data on socioeconomic status and disabling chronic conditions in childhood, published between 1 January 1991 and 31 December 2013.

**Data extraction and synthesis:**

Abstracts were reviewed, full papers obtained, and papers identified for inclusion by 2 independent reviewers. Inclusion decisions were checked by a third reviewer. Where reported, ORs were extracted for low versus high socioeconomic status. For studies reporting raw data but not ORs, ORs were calculated. Narrative analysis was undertaken for studies without data suitable for meta-analysis.

**Results:**

126 studies had data suitable for meta-analysis. ORs for risk estimates were: all-cause disabling chronic conditions 1.72 (95% CI 1.48 to 2.01); psychological disorders 1.88 (95% CI 1.68 to 2.10); intellectual disability 2.41 (95% CI 2.03 to 2.86); activity-limiting asthma 2.20 (95% CI 1.87 to 2.85); cerebral palsy 1.42 (95% CI 1.26 to 1.61); congenital abnormalities 1.41 (95% CI 1.24 to 1.61); epilepsy 1.38 (95% CI 1.20 to 1.59); sensory impairment 1.70 (95% CI 1.39 to 2.07). Heterogeneity was high across most estimates (I^2^>75%). Of the 34 studies without data suitable for meta-analysis, 26 reported results consistent with increased risk associated with low socioeconomic status.

**Conclusions:**

The findings indicate that, in high-income countries, childhood disabling chronic conditions are associated with social disadvantage. Although evidence of an association is consistent across different countries, the review provides limited evidence to explain the association; future research, using longitudinal data, will be required to distinguish low socioeconomic status as the cause or consequence of childhood disabling chronic conditions and the aetiological pathways and mechanisms.

**Strengths and limitations of this study**

- The extensive literature reviewed used a rigorous methodology, and the consistent findings across different country settings suggest the conclusions are robust.
- The high degree of heterogeneity in the pooled estimates represents a threat to their validity; however, the majority of the estimates were robust to sensitivity analysis.
- This review, using both quantitative and qualitative data synthesis, is the first to draw together a large body of studies on the relationship of socioeconomic disadvantage with disabling chronic conditions in childhood in high income countries.

## Introduction

Disabling chronic conditions in childhood are a major global public health issue in high-income as well as low-income countries. Estimates of these conditions in most high-income countries fall between 3.5% and 8.0% of children aged 0–18 years, with some countries reporting that prevalence is increasing.^1^ Although children with these conditions can lead rich and fulfilling lives, many experience poor educational outcomes,^2^ social adversity,^3^ lower levels of social participation^4^ and sometimes pain.^4^ Reducing the prevalence of these conditions in childhood, and the impact on children and their families is, therefore, desirable. Disability is increasingly seen as a ‘dynamic interaction between health conditions and contextual factors, both personal and environmental’,^3^ with social and genetic factors coming together in complex ways to increase a child's risk of developing a chronic disabling condition.^5^

The first World Report on Disability^3^ identifies poverty and socioeconomic disadvantage as possible cause and consequence of disability. This report, however, focuses on adults in developed countries and does not consider the evidence for this association in childhood. Low socioeconomic status (SES) is likely to be both a cause and consequence of disability in childhood, but if and where the low SES sits on the causal pathway remains unclear. Although many studies have examined the association between childhood disabling chronic conditions and SES, to date there has been no published systematic review of studies examining the association in high-income countries. As a result, there is no synthesised evidence on risk, or assessment of the quality of this research. The only available systematic review of literature on this association in low-income and middle-income countries indicated that the evidence was inconsistent and inconclusive, and that many studies had a high/medium risk of bias.^6^

To address this important evidence gap, we undertook a systematic review and meta-analyses of studies in high-income countries to examine the association of SES with childhood disabling chronic conditions. In this paper, the term disabling chronic conditions refers to the range of conditions and impairments lasting at least 6 months that limit a child's normal daily activity. We examine the role of SES in all-cause disabling chronic conditions and in a range of condition groups. As the first systematic review in this area, it provides rigorous evidence on the association between disabling chronic conditions in childhood and SES that will contribute to understanding how to reduce the prevalence and impact of these diverse and complex conditions in childhood.

The main focus of this paper is a quantitative synthesis of the data with meta-analyses of studies that report either ORs or raw data from which these can be estimated. A brief narrative analysis of those studies that could not be entered into meta-analysis is included.

## Methods

### Search strategy and selection criteria

We searched MEDLINE, PsycINFO, ASSIA, EMBASE, Web of Science and EconLit for studies reported between 1 January 1991 and 31 December 2013. For each database, a search strategy using a combination of free text and controlled vocabulary terms was developed (see example in box 1). We used search terms for the exposure of interest (socioeconomic disadvantage) and the outcomes of interest (childhood disabling chronic conditions). Searches of relevant national and international government and non-government organisations’ internet sites were conducted, and reference lists of included studies were screened.

Box 1 **Sample search strategy: Ovid Medline**

1. exp Socioeconomic Factors/(276257)
2. social disadvantage.ab,ti. (370)
3. social deprivation.ab,ti. (722)
4. low income.ab,ti. (11585)
5. social exclusion.ab,ti. (410)
6. lone parenthood.ab,ti. (24)
7. parental disability.ab,ti. (15)
8. 1 or 2 or 3 or 4 or 5 or 6 or 7 (281054)
9. childhood disabilit+ACo-.ab,ti. (201)
10. exp Disabled children/(3092)
11. chronic illness+ACo-.ab,ti. (7432)
12. asthma.ab,ti. (86271)
13. cerebral palsy.ab,ti. (10905)
14. epilepsy.ab,ti. (52945)
15. hearing impairment.ab,ti. (4631)
16. visual impairment.ab,ti. (4265)
17. congenital abnormalit+ACo-.ab,ti. (4285)
18. ((long-term illness+ACo- or long-standing illness+ACo-) and limiting).ab,ti. (126)
19. activity limiting illness+ACo-.ab,ti. (1)
20. exp+ACI-Attention Deficit and Disruptive BehaviorDisorders+ACI-/(17055)
21. emotional disorders.mp.orexp Child Behavior Disorders/epidemiology (1066)
22. +ACo-Mental Retardation/(28678)
23. +ACo-Learning Disorders/(7918)
24. +ACo-Communication Disorders/(875)
25. or/9–24 (224506)
26. 8 and 25 (7275)
27. (addiction+ACo- or addicted or drug taking or smoking).mp. (172219)
28. exp Substance-Related Disorders/(310751)
29. alcohol+ACo-.ab,ti. (176708)
30. +ACo-Substance-Related Disorders/epidemiology or +ACo-Smoking/epidemiology or +ACo-Opioid-Related Disorders/epidemiology or +ACo-Alcoholism/ep (3642)
31. exp HIV/(67947)
32. exp Acquired Immunodeficiency Syndrome/(68867)
33. or/27–32 (665725)
34. 26 not 33 (6435)
35. expcanada/or exp united states/or exp japan/or exp+ACI-republic of korea+ACI-/or expaustralia/or expaustria/or expbelgium/or expczech republic/or exphungary/or exppoland/or expslovakia/or expslovenia/or expfinland/or france/or expgermany/or exp great britain/or expgreece/or expiceland/or exp ireland/or expitaly/or expluxembourg/or expnetherlands/or expportugal/or expdenmark/or expnorway/or expsweden/or expspain/or expswitzerland/or exp new zealand/or exp Israel/(1985719)
36. 34 and 35 (2806)
37. limit 36 to (english language and humans) (2583)
38. limit 37 to yr+AD0AIg-1985 -Current+ACI- (2318)
39. limit 38 to +ACI-all child (0 to 18 years)+ACI- (1514)
40. l/39 ed+AD0-20101123-20110208 (30)

**Figure 1:**
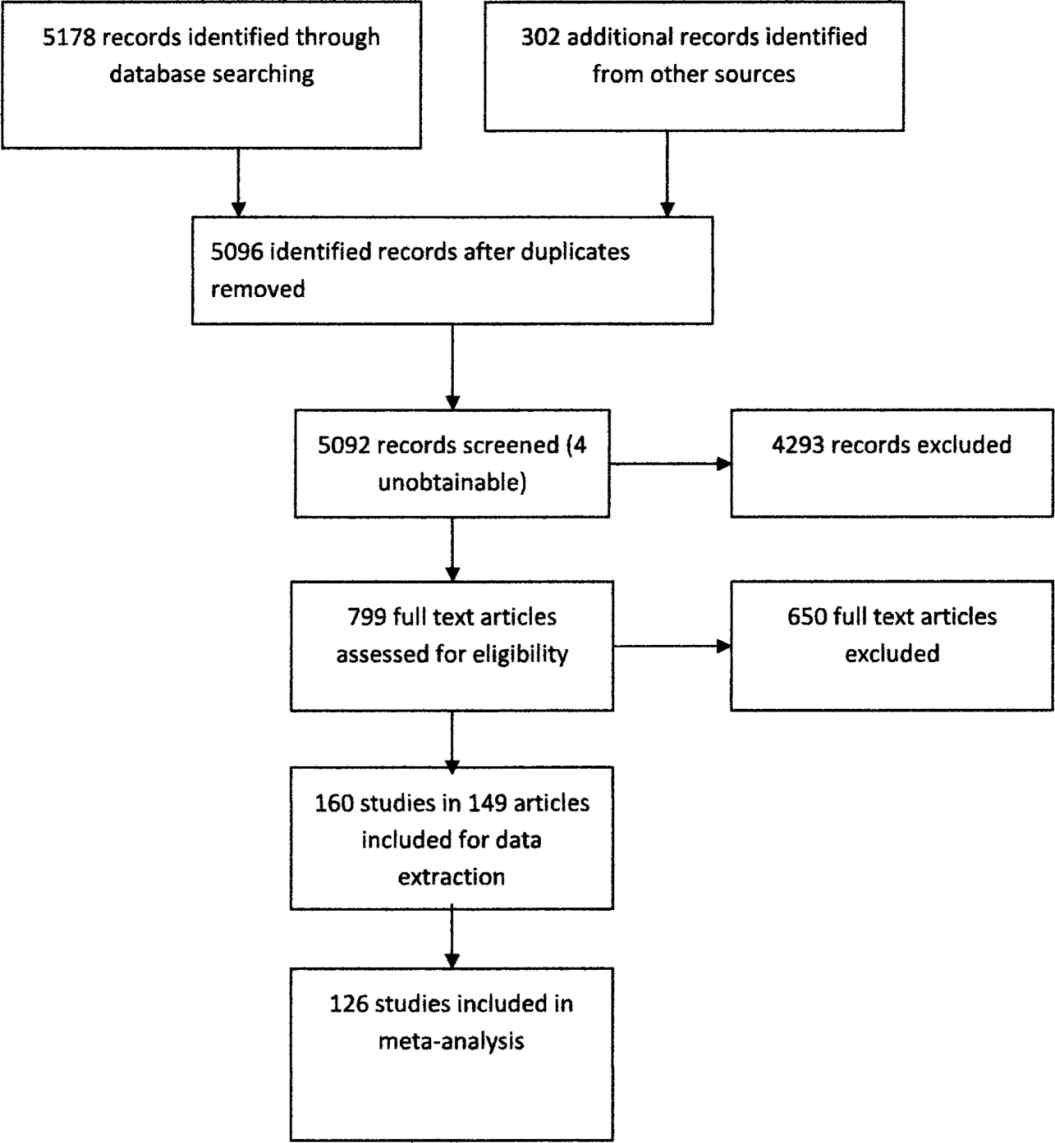
Flow chart of study selection.

We contacted international experts to identify studies not captured in other searches. English language publications only were included. A total of 5480 titles and abstracts, and 799 full-text articles and reports were independently screened by two reviewers (JMR and NJS). Of the full-text articles and reports, data were extracted onto standard forms for potentially relevant studies by one reviewer (NJS) and checked by a second reviewer (CMB; weighted k=0.91). Differences of opinion were resolved in discussion with the third reviewer (JMR). Figure 1 shows the flow chart of study selection.

Studies were included if the design was cross-sectional, case–control, cohort, register-based or based on routinely collected data derived from a whole population and reported empirical, individual level data on the association of SES with disabling chronic conditions in childhood (box 2). Studies that were based on selected populations (eg, inner-city dwellers or minority ethnic groups only) and those not reporting results for children separately from adults were excluded as were those in which the duration or activity limitation of the condition was not specified. We excluded studies in which the disabling chronic condition was reported as a continuous variable.

Box 2 **Inclusion and exclusion criteria**

Inclusion

- Cross-sectional, cohort, case–control studies and those based on condition registers and routine data published between 1 January 1991 and 31 December 2013
- Studies conducted in high-income OECD countries as defined by the World Bank*
- Empirical data on the association of socioeconomic status (SES) with disabling chronic conditions in children reported
- Conditions lasting at least 6 months AND associated with limitation in normal daily activity reported
- Studies based on whole population data or samples representative of whole populations

Exclusion

- Studies published before 1991 and after 2013
- Studies conducted in countries not on the World Bank OECD high-income list*

Reviews

- Association of SES with disabling chronic conditions not reported
- Reported conditions not defined by duration AND activity limitation
- Population subgroups only reported
- Sample not representative of whole population
- Mortality studies
- Studies reporting all age data with no data on children as a separate group
- Studies reporting non-individual level episode data
- Studies reporting outcomes as non-dichotomised, continuous variables
- Studies using aggregated SES measures which include major confounders of the relationship with disabling chronic conditions, such as ethnicity and lone parenthood.

*See World Bank classification of countries by income at http://data.worldbank.org/about/country-and-lending-groups

Studies were assessed for quality by one reviewer (NJS) and checked by a second reviewer (CMB). Differences of opinion were settled by discussion. We extended the Newcastle-Ottawa Scales^7^ to assess risk of bias for each of the five study types: cross-sectional, case–control, cohort, register-based and routinely collected data. Standard criteria for assessing risk of bias for each of the study types are shown in online supplementary appendix 1. Major confounding variables, referred to in online supplementary appendix 1, were child's age, child's sex, race/ethnicity and lone parenthood.

### Types of disabling chronic conditions and SES measures

Children included in the studies had a range of disabling chronic conditions characterised by duration longer than 6 months and associated limitation of normal daily activity. Conditions were grouped as follows: all-cause disabling chronic conditions; psychological disorders; intellectual disability; sensory impairments; congenital abnormalities; specific conditions, such as asthma, cerebral palsy and epilepsy; and a miscellaneous group of conditions with insufficient numbers for entry into meta-analysis (table 1). SES measures were grouped as follows: parental education level, income, occupational class, area-based SES measures, poverty, housing tenure, workless household, composite SES measures, miscellaneous measures (table 2). Low SES was defined as the most disadvantaged group for which prevalence was reported in each study.

**Table 1:** Groups of disabling chronic conditions (DCC) and included conditions

DCC group	Number of included studies	Number of affected children	Conditions included in the group
All-cause disabling chronic conditions	29 studies^1^ ^2^ ^8–30^	76226* affected children	Combined categories of all chronic conditions with associated activity limitation, including physical, sensory and psychological disabilities or long-term health problems
Psychological disorders	72 studies^11^ ^16^ ^18^ ^31–94^	72277* affected children	All-cause psychological disorder, attention deficit hyperactivity disorder, autistic spectrum disorder, emotional disorder, oppositional defiant disorder, conduct disorder, internalising and externalising behaviour problems, obsessive compulsive disorder, chronic fatigue syndrome
Intellectual disability	25studies,^52^ ^60^ ^69^ ^71^ ^84^ ^95–113^	633235* affected children	Children with IQ <70 or equivalent measure—7 of these studies report mild and moderate/severe disability
Sensory impairments	12 studies^11^ ^51^ ^71^ ^84^ ^88^ ^112^ ^114–119^	11994* affected children	Hearing impairment and visual impairment
Cerebral palsy	8 studies^84^ ^120–126^	16084 affected children	Non-acquired and acquired cerebral palsy
Epilepsy	9 studies^11^ ^31^ ^104^ ^112^ ^127–131^	13562* affected children	Recurrent epileptic seizures excluding febrile seizures
Asthma	13 studies^11^ ^51^ ^132–141^	6407 affected children	Asthma with activity limitation and/or asthma requiring hospital admission
Congenital anomalies identifiable at birth	14 studies^110^ ^142–153^	41956* affected children	Neural tube defects compatible with life, cleft lip and palate, other congenital and chromosomal abnormalities
Miscellaneous conditions (insufficient numbers for meta-analysis)	4 studies^85^ ^104^ ^112^ ^154^	8954 children	Crohn's disease, Down's syndrome, diabetes mellitus, heart disease

*Indicates incomplete totals as some studies reported no data on number of participants.

**Table 2:** Socioeconomic status (SES) measures

SES measure group*	Number of studies	Specific measures included in group
Parental education	56 studies	Maternal education; paternal education; highest parental educational level; years of education; qualifications achieved
Income	49 studies	Household income; equivalised household income using OECD method; urban income
Poverty	42 studies	Relationship to Federal Poverty Line (USA); <60% of national median income (UK); receipt of social safety net benefits
Occupational class	37 studies	UK Registrar General's social class; UK National Statistics Socio-economic Classification; other country classifications (Finland, Denmark, Sweden, Holland); Bilshen Occupational Scale (Canada)
Area-based SES measures	35 studies	UK deprivation indices (Townsend; Carstairs); Acorn area classification (UK); census-derived area income measures (USA and Canada); Socio-economic Indicators for Area—SEIFA (Australia); inner city vs suburbs
Housing tenure	11 studies—all UK	Rented vs owner-occupied accommodation
Workless household	9 studies	Households with no working adult
Composite individual level SES measures	7 studies	Winkler index; occupation and education of both mothers and fathers; occupation and education of both parents and household income; social disadvantage index (occupation; housing tenure; car ownership)
Other	8 studies	Material hardship (unable to afford essential items); debt; car ownership; family affluence scale

*Thirty-seven per cent of studies reported more than one SES measure.

OECD, Organisation for Economic Co-operation and Development.

### Data analysis

#### Quantitative data synthesis

We extracted crude or adjusted ORs with 95% CIs by SES measures from studies in which these were reported. Where studies reported a disabling chronic condition by more than one SES measure, we included the measure associated with the highest OR in the initial meta-analysis and undertook sensitivity analysis using the SES measure with the lowest OR. For studies reporting raw data, crude ORs with 95% CIs were calculated for comparison of children with disabilities with children without disabilities. Where results were reported separately for boys and girls, ORs for all children were recalculated from raw data. For studies in which neither ORs nor raw data were reported, the investigators were contacted to request data. Pooled ORs with 95% CIs for the risk of low SES were calculated using a random-effects model using the function for summary meta-analysis in StatsDirect (V.2.7.8). Heterogeneity in pooled data was estimated using the I^2^ statistic and risk of bias using the Egger^155^ and Begg-Mazumdar tests.^156^ Forest plots were generated showing ORs with 95% CIs for each study and the overall random-effects pooled estimate. For pooled estimates with a high I^2^ statistic, sensitivity analyses, aimed at explaining some or all of the heterogeneity, were undertaken by re-running the meta-analyses comparing studies with specific characteristics which were identified a priori as the most likely to contribute to heterogeneity (eg, geographical area of study (the USA vs the rest), studies with high vs medium/low risk of bias; studies reporting crude ORs only versus adjusted ORs; different SES measures used in same study; different age ranges).

### Narrative data analysis

Studies not reporting ORs with 95% CIs or raw data from which these could be calculated were not entered into the meta-analyses. We undertook narrative analysis of these studies. A simple count of studies with results consistent and inconsistent with the pooled estimates was made, and the latter were analysed in more detail.

### Role of the funding source

The funder of the study had no role in study design, data collection, data analysis, data interpretation or writing of the report. All authors had full access to all data in the study and had final responsibility for the decision to submit for publication.

## Results

Our search identified 160 studies with relevant data reported in 149 papers (see figure 1 and online supplementary appendix 2). Ninety studies were cross-sectional, 25 cohort, 21 based on routine data, 16 case–control and 8 based on disease registers (see online supplementary appendix 1). The types of disabling chronic condition reported are listed in table 1. Psychological disorders were the most frequently reported conditions (72 studies) followed by all-cause disabling chronic conditions (29 studies) and intellectual disability (25 studies). A combined total of more than 889 618 children with disabling chronic conditions were included in the studies, in which sample sizes varied between 50 and 41 928 607. All but one study^63^ reported data on both sexes, although data on the association with SES were reported separately for boys and girls in some studies. Ages of the children included in the studies were mainly between 0 and 18 years; 11 included young people aged 19–21 years. Eighty-six studies were carried out in the WHO European region (the UK 55; Finland 8; Denmark 3; Norway 3; Sweden 1; two or more Nordic countries 5; Holland 3; Germany 3; Spain 3; Italy 1; Belgium 1), 63 in the WHO region of the Americas (the USA 53; Canada 10) and 11 in the WHO Western Pacific Region (Australia 9; New Zealand 2).

Risk of bias was low in 13 studies of which 6 were based on routinely collected data, 4 case–control, 1 cohort and 2 register-based studies. No cross-sectional studies had low risk of bias. The majority (58%) had a medium risk of bias and 53 studies had a high risk. Non-adjustment for major confounding variables was the most common source of bias affecting 75% of studies. Outcomes were parent-reported in most cross-sectional and cohort studies with only five cross-sectional and five cohort studies reporting independent blind assessment of the outcome. In the remaining three study types, 67% of cases were independently clinically identified. Insufficient information was given in five of the cross-sectional and cohort studies on the representativeness of the study sample and of the controls in four case–control studies. Denominator populations were clearly defined in all but one of the register-based and routine data-based studies (see online supplementary appendix 1).

### 

#### Quantitative data synthesis

**Figure 2:**
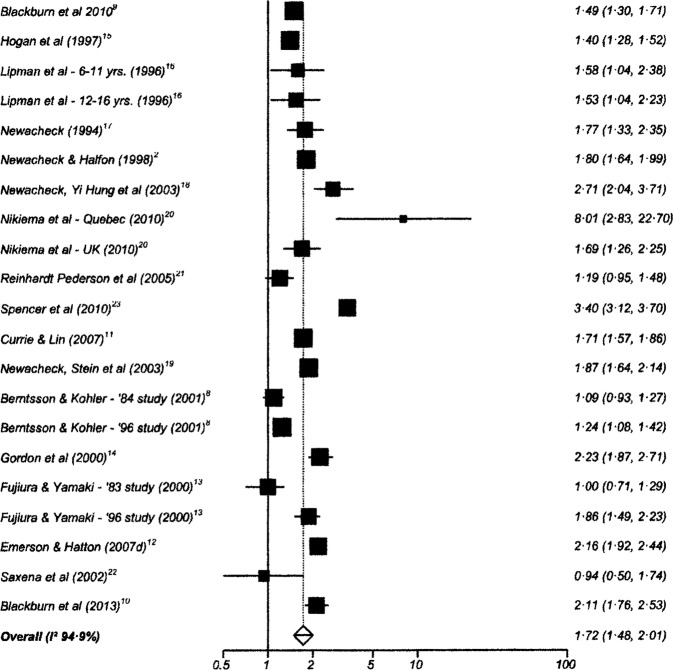
Risk estimates of low socioeconomic status in children with all-cause disabling chronic conditions.

**Figure 3:**
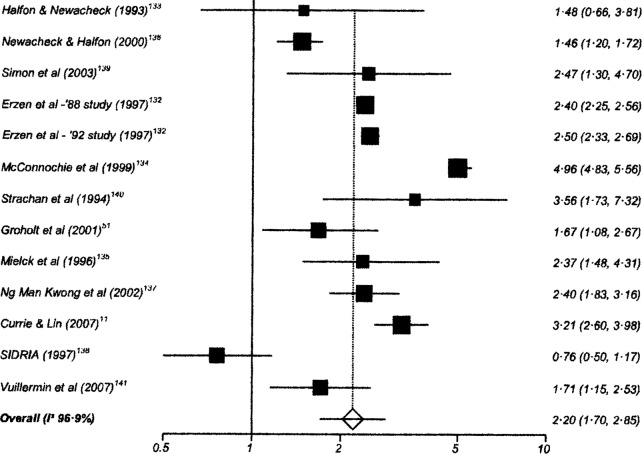
Risk estimates of low socioeconomic status in children with asthma.

**Figure 4:**
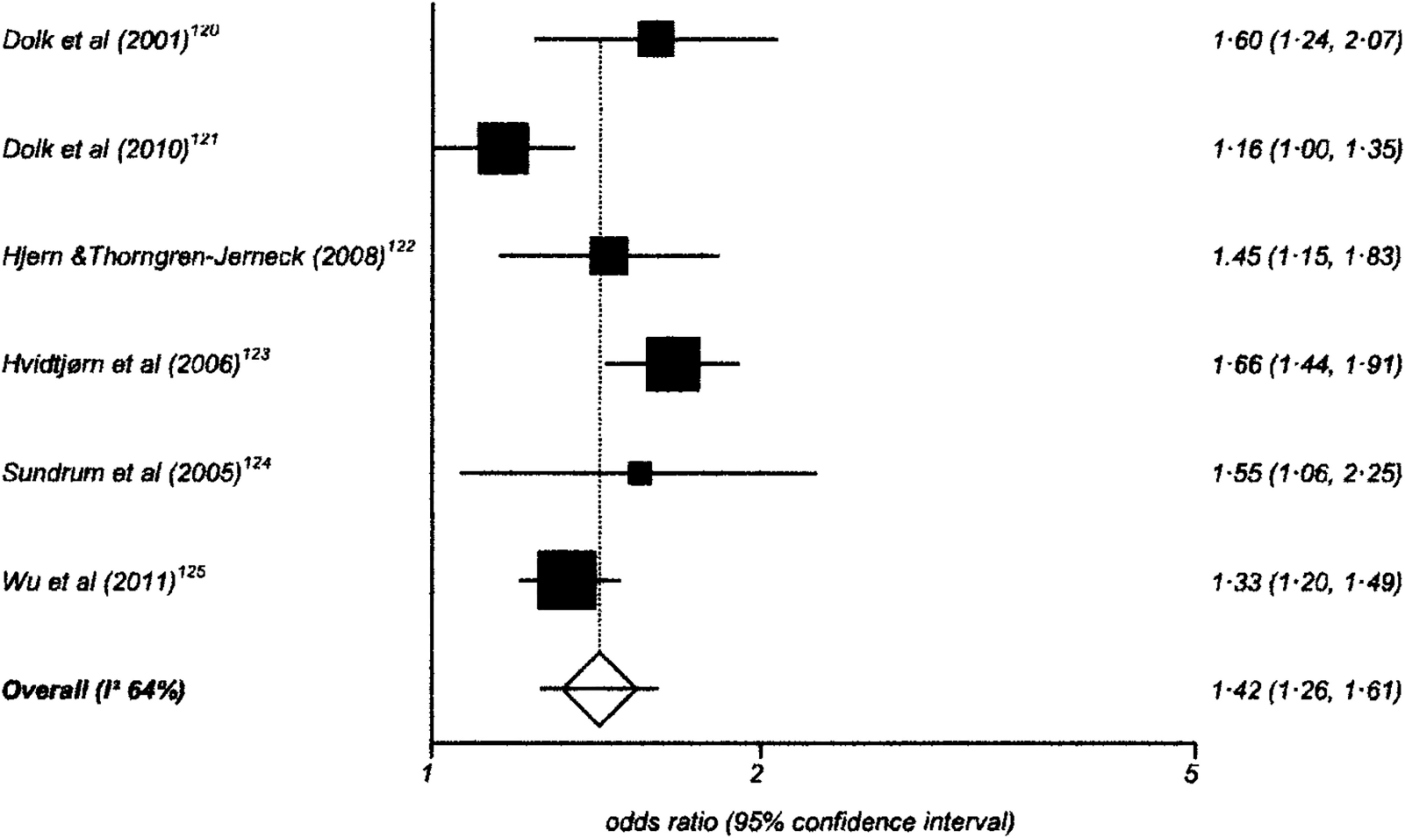
Risk estimates of low socioeconomic status in children with cerebral palsy.

**Figure 5:**
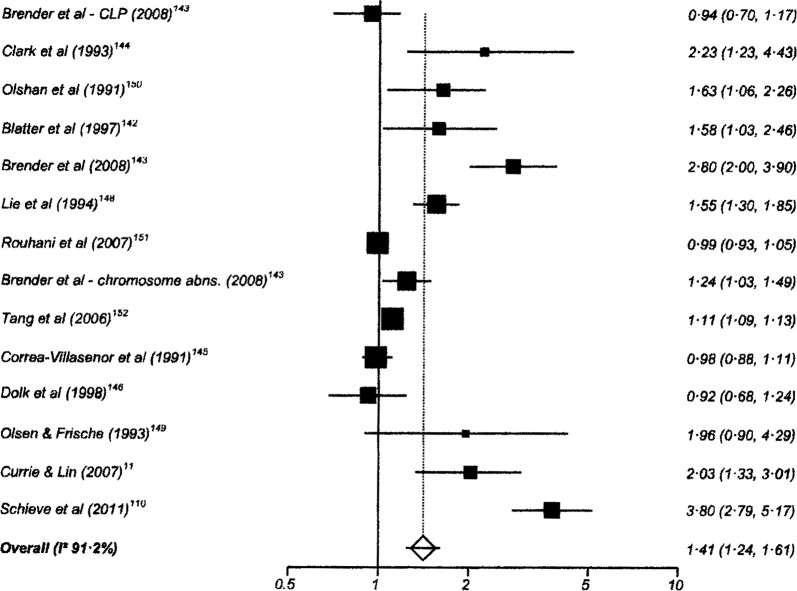
Risk estimates of low socioeconomic status in children with congenital abnormalities.

**Figure 6:**
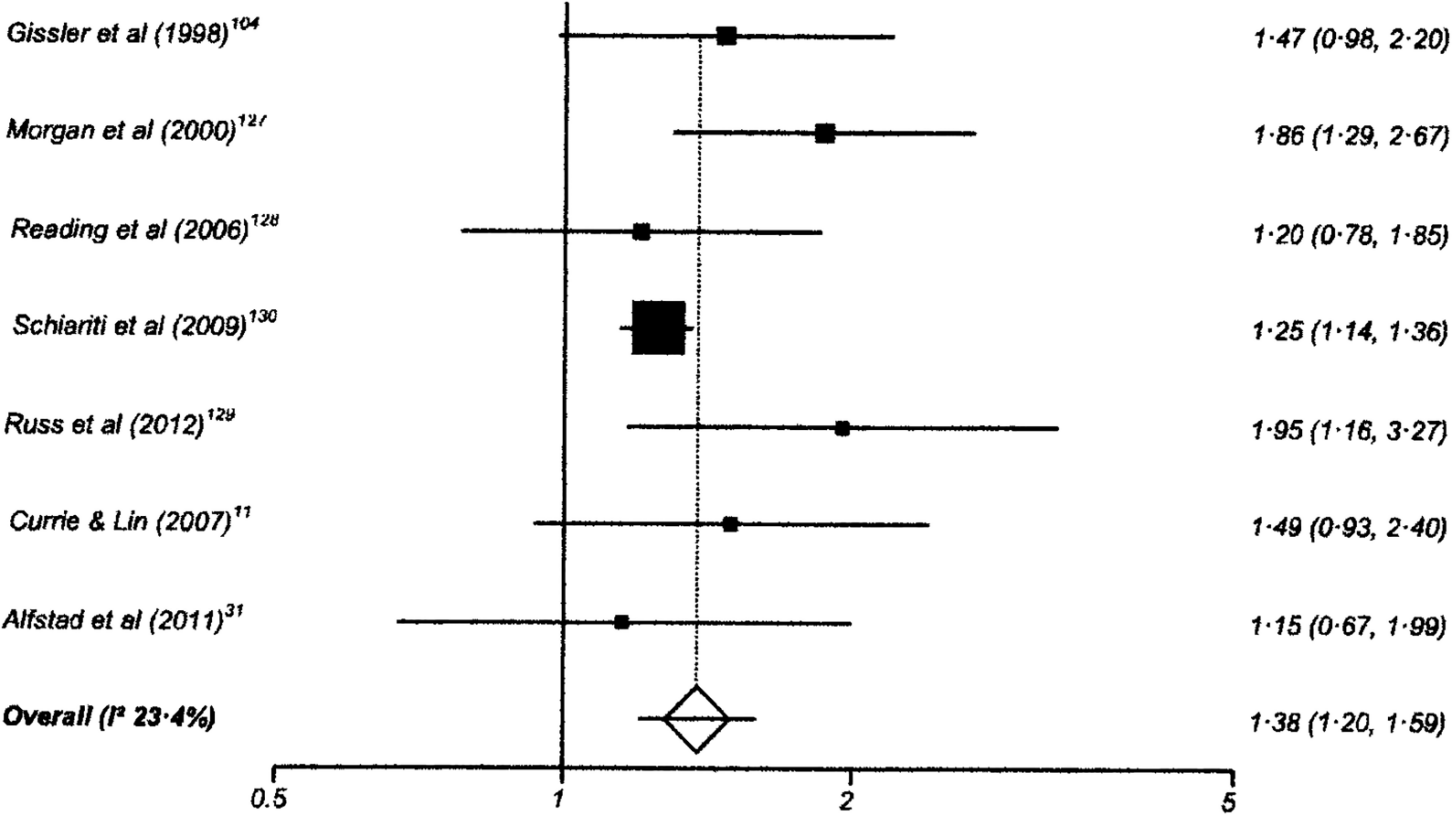
Risk estimates of low socioeconomic status in children with epilepsy.

**Figure 7:**
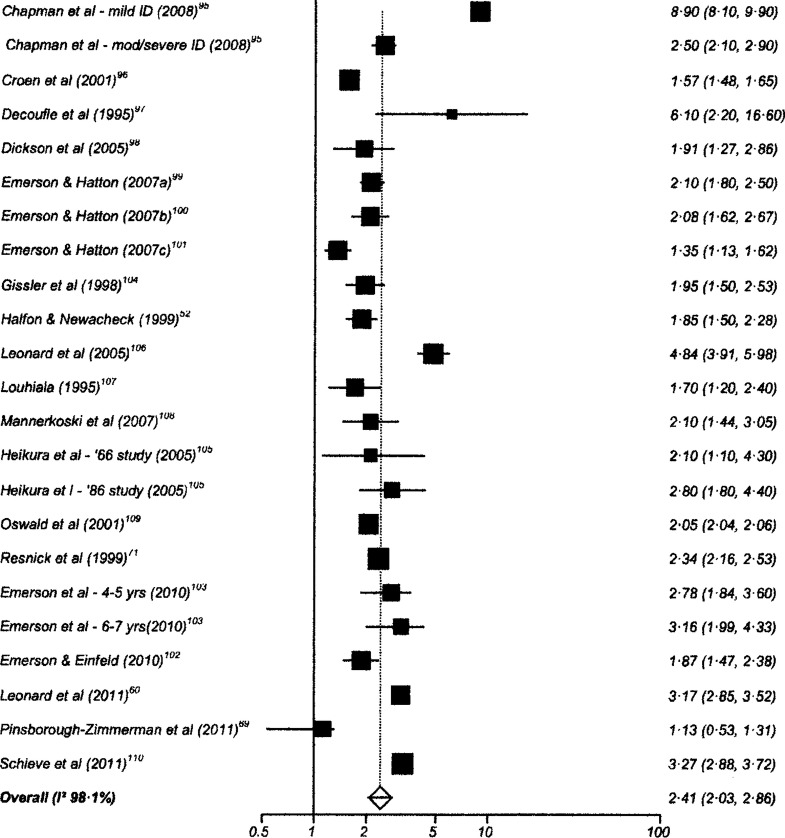
Risk estimates of low socioeconomic status in children with intellectual disability (ID).

**Figure 8:**
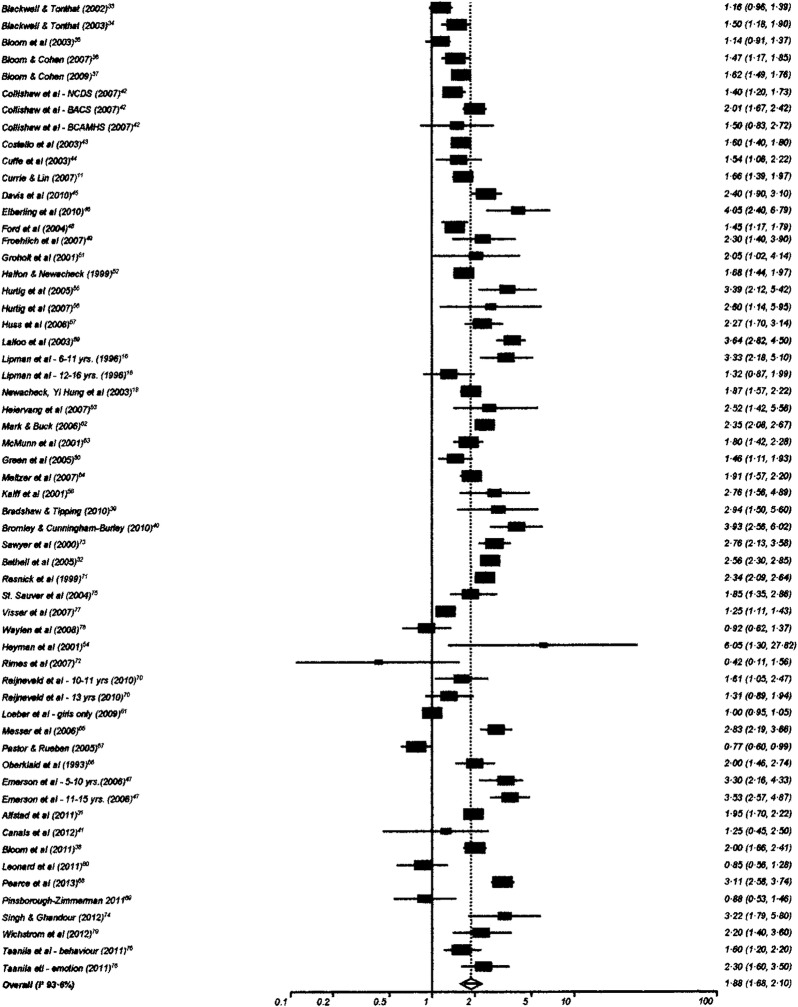
Risk estimates of low socioeconomic status in children with psychological disorders.

**Figure 9:**
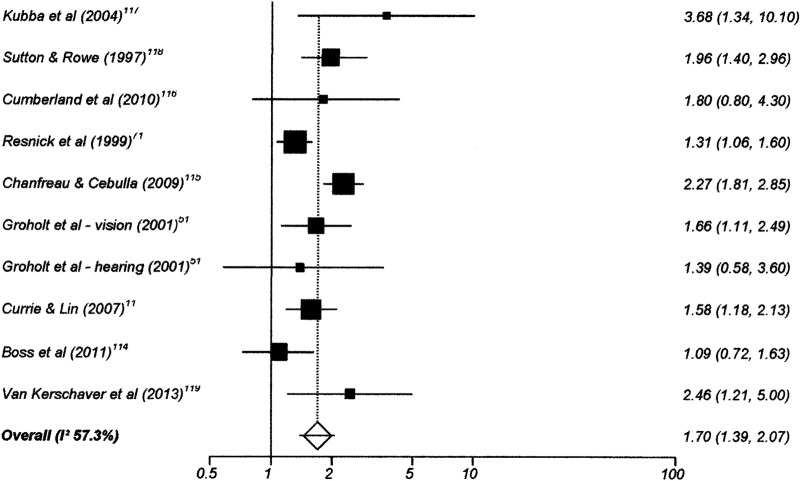
Risk estimates of low socioeconomic status in children with sensory disabilities.

One hundred and twenty-six studies had data suitable for meta-analysis. Risk and pooled random-effects estimates for groups of disabling chronic conditions are shown in figures 2–9 and table 3. The pooled ORs for the different groups of disabling chronic conditions by low SES were as follows: 1.72 for 20 studies reporting all-cause disabling chronic conditions, 1.88 for 55 studies reporting psychological disorders, 2.41 for 21 studies reporting intellectual disability, 2.20 for 13 studies reporting activity limitation or hospital admission for asthma, 1.42 for 6 studies reporting cerebral palsy, 1.41 for 13 studies of congenital abnormalities, 1.38 for 6 studies of epilepsy and 1.70 for 9 studies of sensory impairments. The I^2^ statistic was >75% for all, but the pooled estimates for cerebral palsy, epilepsy and sensory impairments. Pooled estimates were available for specific psychological disorders (attention deficit hyperactivity disorder (ADHD; 1.63 (1.42 to 1.86)), conduct disorder (1.93 (1.58 to 2.38)) and emotional disorder (2.03 (1.67 to 2.47)), and for mild (3.94 (2.26 to 6.86)) and moderate/severe (2.19 (1.84 to 2.64)) intellectual disability (forest plots not shown)).

**Table 3:** Pooled random effects estimates for low socioeconomic status by groups of disabling chronic conditions

Disabling chronic condition	Studies	OR (95% CI)	Heterogeneity (I^2^ statistic)
All-cause disabling chronic conditions	20	1.72 (1.48 to 2.01)	95.0% (94.7% to 95.7%)
Psychological disorders	55	1.88 (1.68 to 2.10)	93.6% (92.6% to 94.3%)
Intellectual disability	21	2.41 (2.03 to 2.86)	98.1% (97.9% to 98.3%)
Activity-limitation or hospital admission for asthma	13	2.20 (1.87 to 2.85)	96.9% (96.2% to 97.4%)
Cerebral Palsy	6	1.42 (1.26 to 1.61)	64.0% (0% to 83.1%)
Congenital abnormalities	13	1.41 (1.24 to 1.61)	91.2% (87.6% to 93.4%)
Epilepsy	6	1.38 (1.20 to 1.59)	23.4% (0% to 67.5%)
Sensory impairment	9	1.70 (1.39 to 2.07)	57.3% (0 to 77.2%)

Bias indicators were non-significant for all groups of disabling chronic conditions except for psychological disorders for which the Egger test was significant (p=0.0012) but not the Begg-Mazumdar test (p=0.97). Sensitivity analyses showed no significant differences in pooled estimates based on specific characteristics of studies for all disabling chronic condition groups except asthma and psychological disorders (see online supplementary appendix 3). The pooled OR for asthma for studies reporting crude ORs only (3.00 (95% CI 2.89 to 3.11)) was significantly higher than that for those reporting adjusted ORs (1.75 (95% CI 1.35 to 2.36)). For psychological disorders, the pooled OR for studies including only children <12 years of age was significantly higher than that for studies including older children (pooled ORs 2.48 (95% CI 2.07 to 2.97) and 1.77 (1.55 to 2.03), respectively).

#### Narrative data analysis

Data from 34 studies were not suitable for meta-analysis. Of the eight studies reporting all-cause disabling chronic conditions, three reported findings suggesting no association with low SES. Ford *et al*,^26^ based on data from the baseline study of 15 year-olds included in the prospective West of Scotland Twenty-07 study, report an association of limiting long-standing illness among boys with low parental social class (p<0.05), but not for girls. In a review article, West^29^ reports data on 18 year-olds from the West of Scotland Twenty-07 study showing no significant differences in prevalence of all limiting long-standing illness among either males or females by social class, but a higher prevalence among low social class boys of severe limiting long-standing illness (defined as ‘quite a lot’ or ‘very great deal’ of restriction), but not among girls. These studies both carry a high risk of bias (see online supplementary appendix 1) and the reporting of parental social class by young people may not be reliable. West and Sweeting^30^ reported little evidence of SES differences in limiting long-standing illness among boys and girls aged 11, 13 and 15 years in the West of Scotland 11–16 Cohort study. This study used a number of SES measures reported by parents; however, it has a high risk of bias.

Five of the studies not suitable for meta-analysis reporting on psychological disorders found no association with low SES. A reverse SES gradient for ADHD is reported by Hoffman *et al*^87^ based on a cohort initially designed to study the association between exposure to tetrachloroethylene (PCE)-contaminated public drinking water and the risk of reproductive and developmental disorders. Exclusions from the original cohort plus no information about the attrition rate may have led to a non-representative sample for this analysis. The study has a high risk of bias. Khanam *et al*^88^ found no significant association of low income with ADHD among 8-year-old children enrolled in the kindergarten cohort of the Longitudinal Study of Australian Children. The regression models included many socially related variables that could be mediators of the low-income ADHD, thus suggesting the possibility of overcontrolling for SES. The study has a medium risk of bias. No association of behavioural problems at 3 years of age with SES was reported by Sonuga-Barke *et al*^93^ from a cross-sectional study based on developmental clinics run by family doctors in an area of south England. In addition to having a high risk of bias, low SES families may have been under-represented in the sample due to socially patterned differential uptake of developmental checks at 3 years. Boyle *et al*^84^ reported no significant difference in prevalence of ADHD among children aged 3–17 years by poverty or low maternal education. This study, which has a medium risk of bias, was based on a large, aggregated sample from the US National Health Interview Surveys (NHIS) for the years 1997–2008. Blackwell and Tonthat,^33^ and Bloom *et al*^35^ reporting on samples from the NHIS for the years 1998 and 2001, respectively, also showed no association with poverty or parental education, although significant associations were noted for the years 1999^34^ and 2006.^36^

Khanam *et al*^88^ and Boyle *et al*^84^ reported no association of sensory impairments with low SES. As indicated above, the inclusion of many socially related variables in the regression model in Khanam's paper may have been overcontrolled for SES. By contrast, Boyle *et al*^84^ did not control for potential confounders. Boyle *et al*^84^ also reported no association of cerebral palsy with either poverty or maternal education. There were no studies unsuitable for meta-analysis of intellectual disability or congenital abnormalities that reported no association of the outcome with low SES. All studies of asthma had data suitable for meta-analysis.

### Discussion

This is the first systematic review and meta-analyses of studies reporting on the relationship between childhood disabling chronic conditions and low SES in high-income countries. The results of the meta-analyses show that a range of childhood disabling chronic conditions are associated with low SES.

The review shows the association of the most common childhood disabling chronic conditions with low SES. Psychological disorders and intellectual disabilities are among the most common and intractable conditions, and impacts on children, their families and health, social and education services are substantial. The odds of these being reported among low SES households are around twice those for high SES households.

Asthma is one of the most prevalent chronic conditions in childhood in high-income countries. A recent systematic review and meta-analysis reported low SES associated with a higher prevalence of asthma in 63% of studies with a pooled estimate of 1.38 (95% CI 1.37 to 1.39).^157^ We included only studies reporting on asthma severe enough to cause activity limitation and/or hospital admission, and found a strong association with SES. To date, the evidence on the association of cerebral palsy and epilepsy with low SES has also been unclear, and likely to be related to study methodologies.^124^ ^128^ Pooled estimates for both in this review, however, support a significant association.

Confirmation of the association of disabling chronic conditions with low SES using systematic review methodology and generation of pooled estimates of risk is important. Further research, however, is needed to explain this association in high-income countries. One possible explanation is that poor social and environmental conditions in pregnancy and early childhood are on the causal pathway to childhood disabling chronic conditions. Some included papers discuss the poor social conditions that might lead to conditions, such as activity-limiting asthma,^133^ and the role of socially patterned problems in pregnancy in the aetiology of cerebral palsy^120^ and congenital abnormalities.^145^ ^149^ Questions of causality, however, can only be addressed using cohort studies with low risk of bias. The majority of studies in this review were cross-sectional. Of the 21 cohort designs we identified, only 1 was assessed as having a low risk of bias. Low parental education is likely to precede the onset of a child's disabling condition and its association with a range of conditions (see online supplementary appendix) lends support to the explanation that SES is on the causal pathway. The reverse causation explanation is that caring for a child with a disabling chronic condition leads to low SES by limiting household income and increasing household costs. Anderson *et al*^158^ show the impact on family finances and work of having a child with intellectual and/or developmental disability.

The review has several methodological issues and limitations which should be considered when interpreting the findings. Definitions of disability vary widely as do measures used to identify those with disabilities in populations. In line with the focus of the World Report on Disability^3^ and International Classification of Functioning, Disability and Health,^159^ we only included in this review studies reporting on conditions that were both long term and activity limiting. Therefore, studies which used broader definitions of disability were excluded, possibly limiting the scope of the review. The use of expanded MeSH terms for SES may have led to studies being missed; however, the SES measures identified (table 2) include a comprehensive range. There is no internationally agreed definition of ‘low SES’, as different measures are required for different purposes and are meaningful in particular national contexts. The included studies, therefore, use a variety of SES measures and this may be one of the factors contributing to the high level of heterogeneity in the pooled estimates. In sensitivity analyses, however, pooled estimates did not change significantly when different SES measures were used. Many included studies also had a high risk of bias and this is also likely to have contributed to heterogeneity. In particular, in some studies, the failure to adjust for potential confounding factors may have resulted in overestimation of the strength of the association. This was supported by the sensitivity analysis for severe asthma that showed a significantly higher pooled estimate for studies that did not adjust for confounders compared with those that did. There were no similar findings, however, for other conditions. As the sensitivity analyses explain little of the heterogeneity, it is likely that factors we have not been able to measure are responsible. In addition, different sources of information are used to identify a child as having a disabling chronic condition and this may also have contributed to the heterogeneity in the pooled estimates.

This systematic review and meta-analyses make an important contribution to knowledge of the association of childhood disabling chronic conditions with socioeconomic disadvantage in high-income countries. Although caution should be exercised in interpreting the findings due to unexplained heterogeneity and the high risk of bias in many studies, the review indicates that these challenging conditions are more prevalent among children in disadvantaged households in a range of high-income countries. While explanations about the causes of this association are to be found in the literature, further high-quality research in cohort studies with adequate sample sizes is required to more fully address the aetiology of the associations identified by this review.

Our findings have implications for social, economic and health policy. The higher prevalence of these conditions among socioeconomically disadvantaged children in richer nations with very different policy environments represents a major challenge to governments seeking to reduce health inequalities and promote the rights of disabled children. Reducing the association between socioeconomic disadvantage and disabling chronic conditions in childhood is likely to require multidimensional strategies. These might include those proposed in the WHO report on social determinants of health that aim to reduce socioeconomic disadvantage in the early years,^160^ as well as policies that ensure that households with children with disabling chronic conditions have adequate financial support and access to health, education and social care services to meet their needs.
